# Coping with a changing environment: the effects of early life stress

**DOI:** 10.1098/rsos.160382

**Published:** 2016-10-05

**Authors:** Marco A. Vindas, Angelico Madaro, Thomas W. K. Fraser, Erik Höglund, Rolf E. Olsen, Øyvind Øverli, Tore S. Kristiansen

**Affiliations:** 1 Uni Environment, Uni Research AS, Bergen, Norway; 2 Department of Biosciences, University of Oslo, Oslo, Norway; 3 Institute of Marine Research, Matredal, Norway; 4 Department of Production Animal and Clinical Sciences, Norwegian University of Life Sciences, Oslo, Norway; 5 Department of Food Safety and Infection Biology, Norwegian University of Life Sciences, Oslo, Norway; 6 National Institute of Aquatic Resources, Technical University of Denmark, Hirtshals, Denmark; 7 Norwegian Institute for Water Research (NIVA), Oslo, Norway; 8 Department of Biology, Norwegian University of Science and Technology, Trondheim, Norway

**Keywords:** serotonin, neurochemistry, catecholamines, phenotypic plasticity, Atlantic salmon

## Abstract

Ongoing rapid domestication of Atlantic salmon implies that individuals are subjected to evolutionarily novel stressors encountered under conditions of artificial rearing, requiring new levels and directions of flexibility in physiological and behavioural coping mechanisms. Phenotypic plasticity to environmental changes is particularly evident at early life stages. We investigated the performance of salmon, previously subjected to an unpredictable chronic stress (UCS) treatment at an early age (10 month old parr), over several months and life stages. The UCS fish showed overall higher specific growth rates compared with unstressed controls after smoltification, a particularly challenging life stage, and after seawater transfer. Furthermore, subjecting fish to acute stress at the end of the experiment, we found that UCS groups had an overall lower hypothalamic catecholaminergic and brain stem serotonergic response to stress compared with control groups. In addition, serotonergic activity was negatively correlated with final growth rates, which implies that serotonin responsive individuals have growth disadvantages. Altogether, our results may imply that a subdued monoaminergic response in stressful farming environments may be beneficial, because in such situations individuals may be able to reallocate energy from stress responses into other life processes, such as growth.

## 1. Background

Stressful stimuli, over prolonged periods of time, have often been associated with maladaptive behaviour and disease [1]. However, a growing body of evidence indicates that an individual's phenotypic plasticity is highly associated with specific environmental contexts. Therefore, phenotypic plasticity may be defined as, *the individual's capacity to change its phenotype in response to environmental cues in order to increase its fitness in a given environment* [2]. In this context, stressful situations will affect individuals depending on how they are programmed to cope with their environment and this programming may be genetic or acquired during early life stages [3–5]. It has been proposed that a mismatch between the historic and current environment may lead to normally adaptive responses over-riding self-correcting tendencies of emotional mechanisms, and this leads to pathologies [6]. However, early life stress may balance this environmental mismatch by preparing individuals to adaptively cope with a future harsh environment [7]. Atlantic salmon (*Salmo salar*) strains have gone through a rapid and intense domestication in recent years. This involves subjecting salmon to a series of aquaculture environment challenges, which represent stressors that do not necessarily resemble those occurring in nature [8]. Faced by environmental factors for which natural selection has not prepared them, animals undergoing rapid domestication may suffer the aforementioned mismatch between the ancestral and the current environment. However, in agreement with reports from the mammalian literature, it may be possible to prepare individuals to cope with challenges encountered in artificial rearing through environmental programming during early life stages [9,10].

Here, we explore how a chronic stress regime during early rearing of Atlantic salmon affects performance in subsequent life following the juvenile freshwater stage: after smoltification, a major life-history event whereby individuals become adapted to life in seawater and one month after seawater transfer. We hypothesize that individuals repeatedly exposed to stressful stimuli will be able to cope better with subsequent aquaculture stressors compared with less exposed groups. We collected biometric data at several critical time-points and analysed monoamine neurochemistry to determine stress reactivity in the hypothalamus and brain stem (containing important monoaminergic nuclei innervating large parts of the brain [11]), at basal and acute-stress conditions at the end of the experiment. Subsequently, we report for the first time, to our knowledge, long-term effects in monoaminergic regulation following an early life unpredictable chronic stress (UCS) regime in salmonid fish.

## 2. Material and methods

### 2.1. Experimental animals and facilities

Atlantic salmon eggs (Aqua Gen strain, Aqua Gen AS, Trondheim, Norway) were hatched and reared at the Institute of Marine Research (IMR), Matre, Norway. Prior to the experiment, fish were kept in one 10 000 l outdoor tank under standard hatchery conditions with a natural photoperiod (60° N) and temperature regime (approximately 9°C). A month before the start of the experiment, 744, 10-month old fish (average mass 63 g) were randomly transferred into six indoor tanks (400 l; density: 7 kg fish per tank) supplied with flow-through freshwater. Fish were kept at 12°C on a 12 : 12 photoperiod with a water flow of 15 l min^−1^ and maintained on 92% oxygen saturation. Fish were fed with dry pellets (2 mm Skretting Nutra Olimpic, Stavanger, Norway) that were distributed ad libitum three times a day with automatic feeders (Arvo-tec feeding units: Arvo-Tec T drum 2000, Huutokoski, Finland). Tank conditions were monitored and regulated by a fully automated system (SD Matre, Normatic AS, Nordfjordeid, Norway).

### 2.2. Experimental procedure

At the beginning of the experiment, tank groups were randomly assigned to one of two treatments (three replicates per treatment, 124 fish per tank), UCS or no stress (control). The UCS treatment consisted of stressing fish three times per day (at 8.30, 13.00 and 17.00) using eight different stressors in a random and unpredictable order throughout the week (table 1) for a total of three weeks, following the protocol previously described in Madaro *et al*. [12]. Control fish were only subjected to routine practices of tank maintenance, but otherwise left undisturbed. The three times per day feeding distribution was maintained throughout the experiment and was given approximately 1 h after stressors. Importantly, throughout this period fish were sequentially sampled terminally (*n* = 50) in order to quantify their stress response through this period. These data were previously reported by Madaro *et al*. [12]. At the end of the stress regime, all fish were mildly sedated by submerging them in a MS-222 (metacaine) bath (25 mg l^−1^, Finquel®vet, ScanAqua AS, Årnes, Norway, buffered with 25 mg l^−1^ sodium bicarbonate), fork length and body weight recorded and a PIT-tag was inserted into the abdominal cavity for individual recognition (i.e. sampling 1). From this point and until the end of the experiment, all treatment groups were treated equally. After sampling 1, the remaining fish were distributed into two tanks/treatment (111 fish per tank were distributed into the same 400 l tanks described previously, i.e. a density of approximately 7 kg fish per tank) and maintained for six weeks under constant light, fed ad libitum and went through the parr-smolt transformation, which prepares them for the saltwater environment. At the end of this period, fish were mildly sedated as explained above, measured and weighed (i.e. sampling 2). Subsequently, fish were distributed back into three tanks per treatment (74 fish per tank were distributed into the same 400 l tanks described previously), in order to maintain a similar density as we had at the start of the experiment (approximately 7 kg fish per tank). At this point, the water flow was switched into full strength seawater (35 ppt) for a period of four weeks before the final sampling (i.e. sampling 3).

**Table 1.** Description of stressful stimuli used during the stress treatment. Stressors were given three times per day during 23 days. Three stressor types were randomly chosen daily in order to maintain unpredictability. Modified from Madaro *et al.* [12].

stressful stimuli	elapsed time	methodology
hypoxia	5 min	lowering the water's oxygen saturation to 40% by closing the intake of water flow
low water level	5 min	lowering water level to a total of 3 cm depth while maintaining a constant flow of water
cold shock	120 min	decreasing the water temperature from 12°C to 4°C
heat shock	120 min	increasing the water temperature from 12°C to 19°C
aberrant noise	5 min	hitting the tank repeatedly with a metal bar
flashing light	5 min	subjecting all fish to an intermittent flashing light under total darkness (i.e. ambient lights were turned off)
chasing	5 min	using a net to stir the tank simulating a chase
netting and air exposure	3 min	netting fish and exposing them briefly to air (±1 s) before release

### 2.3. Final sampling protocol

**Figure 1.**
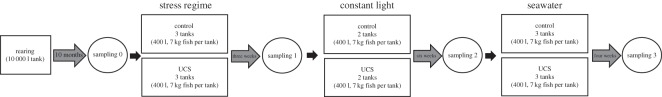
Schematic representation of the experimental protocol. UCS, unpredictable chronic stress.

During the final sampling (i.e. sampling 3), a total of 120 fish were sampled at either basal or acute-stress conditions (*n* = 30 per treatment per condition). The acute-stress challenge consisted of collecting fish with a net and exposing them to air for 15 s and a subsequent confinement test in a 10 l bucket for 5 min. Fish were then left to recover for 1 h before sampling in a 400 l tank (approximately 1.6 kg fish per tank). All fish (stressed and non-stressed) were euthanized with an overdose of MS-222 (1 g l^−1^) which rendered them completely motionless (no opercular movement) within 10 s of immersion. Fish were rapidly weighed, fork length measured and decapitated for brain dissection. The brain stem and hypothalamus were quickly excised within 2 min, snap-frozen in liquid nitrogen and stored at −80°C for later analysis. For a schematic representation of the experimental protocol please refer to figure 1.

### 2.4. The specific growth rate and condition (*K*) factor

The per cent of body weight gain per day may be studied by calculating the specific growth rate (SGR), which allows for comparison of growth rate and fish weight in a linear manner by correcting for fish size effects. This is done by using the formula (2.1):

2.1 $$\text{SGR}=\left[\frac{\left(\text{log}\;W_{2}-\text{log}\;W_{1}\right)}{\left(t_{2}-t_{1}\right)}\right]\times 100,$$

where *W*_1_ and *W*_2_ are the weight (g) at the start (*t*_1_) and end (*t*_2_) of the specific growth period of interest [13].

The SGR for individual fish was calculated between samplings 1 and 2 and between samplings 2 and 3. The first SGR value in figure 1*c* is based on the mean weight/treatment between sampling 0 and sampling 1, because prior to sampling 1, the fish had not been individually tagged. This SGR value is used to illustrate a general tendency, but was not included in the statistical analysis of the data.

Fulton's condition factor, *K* factor, was calculated in order to standardize the assessment of fish nutritional status. Typically, within a population a low *K* level of less than 0.9 indicates low performance and overall emaciation (low lipid reserve levels), while values above 1 indicate high lipid reserves and suggests good health [14,15]. The *K* factor is calculated by using the following formula (2.2):

2.2 $$K=\left(\frac{W}{L^{3}}\right)\times 100,$$

where *W* is the weight (g) and *L* is the fork length (cm) of the fish.

The *K* factor was calculated for each sampling period. That is, individual weight and length values were used at each sampling (1, 2 and 3) in our calculations in order to obtain specific values at each time point.

### 2.5. Brain monoamine neurochemistry

Frozen brain stems and hypothalamus were homogenized in 4% ice cold per chloric acid (PCA) containing 0.2% EDTA and 3,4-dihydroxybenzyl amine hydrobromide (DHBA, 40 ng ml^−1^) as an internal standard using either a Potter–Elvehjem homogenizer or an MSE 100 W ultrasonic disintegrator, respectively. After spinning samples for 10 min at 15.493 rcf and 4°C, the supernatant was analysed by means of high-performance liquid chromatography (HPLC). The mobile phase was made up of 12 µM EDTA, 86 mM sodium phosphate and 1.4 mM sodium octyl sulfate in deionized water (resistance 18.2 MW), containing 7% acetonitrile set to pH 3.1 using phosphoric acid. The system contains a solvent delivery system (Shimadzu, LC-10AD), an auto-injector (Famos, Spark), a reverse phase column (4.6 mm 100 mm, Hichrom, C18, 3.5 mm) and an ESA Coulochem II detector (ESA, Bedford, MA, USA) with two electrodes at −40 mV and +320 mV. A conditioning electrode with a potential of +40 mV was used to oxidize possible contaminants before analysis. Brain stem concentrations of serotonin (5-hydroxy-tryptamine; 5-HT), dopamine (DA), norepinephrine (NE) and their corresponding catabolites 5-hydroxyindoleacetic acid (5-HIAA), 3,4-dihydroxyphenylacetic acid (DOPAC) and 3-methoxy-4-hydroxyphenylglycol (MHPG) were quantified by comparison with standards and corrected for recovery of the internal standard using HPLC software (CSW, Data Apex Ltd, The Czech Republic). Owing to extraction and processing problems, 22 control samples from the hypothalamus were lost (11 at basal and 11 post-stress). In addition, MHPG levels were below detection level in five control and three UCS samples at basal levels in the brain stem and in three UCS fish in the hypothalamus at basal conditions. Therefore, these individuals were given the lowest detected value.

### 2.6. Statistical analyses

R v. 3.2.3 (R Development Core Team, http://www.r-project.org) and the statistical packages ‘nlme’ and ‘MuMIn’ were used for linear models (LM) and linear mixed effect models (LME). Body weight, SGR and *K* factor values for samplings 1–3 were analysed by LME with treatment (stress regime) and sampling time as categorical independent variables and fish identification as the random effect. Weight data were missing from 15 control fish at sampling time 2; therefore, these individuals were not included in the growth and body size analysis. A separate LM with only treatment as the independent variable was used to analyse weight and *K* factor between groups at the start of the experiment (sample 0). LME models were also used for all monoaminergic neurochemistry data, with treatment (stress regime) and stress (basal conditions versus acute stress) as categorically independent variables, and tank as a random effect. The initial LME models allowed the independent variables to interact, i.e. treatment × time for body size/growth data or treatment × stress for neurochemistry. However, the final model was selected based on a comparison of all possible model combinations, with the final model being the one with the lowest Akaike information criterion (AICc) score, i.e. the best data fit. Where significant interaction effects were observed, contrast values were used to identify effects within sampling time for growth/body size data, or treatment/stress groups for brain neurochemistry. An examination of the residual plots made sure that there were no systemic errors within the residuals of the final models. In some instances, data were log^e^ transformed to improve data fit as judged by examination of residual plots. For the hypothalamus, two individuals had 5-HIAA/5-HT and DOPAC/DA ratios that were outside the mean (i.e. more than 5 s.d.) of all the remaining fish, one UCS basal fish and one control stressed fish. These two individuals were considered as statistical outliers and removed from the analysis. Spearman's correlation analysis was used to analyse the relationship between the brain stem and hypothalamic serotonergic activity and the final SGR (at sampling 3). Significance was assigned at *p* < 0.05.

## 3. Results

### 3.1. Body weight and condition

**Figure 2.**
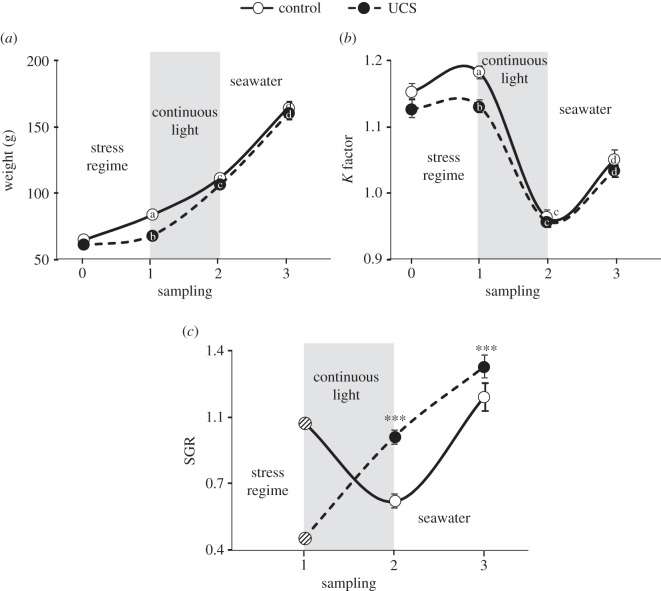
Mean (±s.e.m.) body weight (*a*), *K* factor (*b*) and specific growth rate (SGR) (*c*) of unpredictable chronic stress (UCS) and control Atlantic salmon groups at start of the experiment (sample 0, *n* = 15/treatment), following unpredictable chronic stress (sample 1, *n* = 57 for control and 60 for UCS), after smoltification triggered by continuous light (sample 2, *n* = 45 for control and 60 for UCS) and after transfer into seawater (sample 3, *n* = 60 per treatment). Note that at sampling 0 fish were not individually marked, therefore the SGR mean for sampling 1 was calculated after pooling body weight values for individuals in each treatment group at the start and end of the stress regime. Importantly, this was only done in order to illustrate the general tendency in treatment groups, but *was not* included in statistical analysis. Lowercase letters indicate a significant linear model effect (LME, *p* < 0.01) treatment effects within time point, and asterisks indicate a treatment effect in panel (*c*). Body weight: treatment *t*_118_ = −6.82, *p* = 0.001, time *t*_220_ = 33.58, *p* < 0.001, interaction *t*_220_ = 6.54, *p* < 0.001; *K* factor: treatment *t*_118_ = −2.99, *p* = 0.003, time (sampling 2) *t*_218_ = −16.43, *p* < 0.001, time (sampling 3) *t*_218_ = −10.01, *p* < 0.001, treatment × time (sampling 2) *t*_218_ = 2.74, *p* = 0.007, treatment × time (sampling 3) *t*_218_ = 2.05, *p* = 0.041 SGR: treatment *t*_103_ = 6.2, *p* < 0.001, time *t*_103_ = 7.7, *p* < 0.001, interaction *t*_103_ = −1.7, *p* = 0.09. The contrast value for all statistics is control fish at time 1 (sampling 1).

Fish groups did not differ in body weight (*t*_28_ = −1.39, *p* = 0.18; mean: 63 ± 1 and 63 ± 2 for UCS and control, respectively) or condition (*t*_28_ = −1.53, *p* = 0.14; mean: 1.15 ± 0.01 and 1.18 ± 0.01 for UCS and control, respectively) at the start of the experiment (sample 0). Thereafter, the UCS group had a significant lower body weight and condition following the stress treatment, compared with controls but no effect immediately after smoltification or after one month in seawater (figure 2).

### 3.2. Specific growth rate

We were unable to calculate individual SGR values after the stress regime (sampling 1), as fish had not been individually marked at the start of the experiment (sampling 0). However, pooling weight values for individuals in each treatment group at the start and end of the stress regime illustrates the general tendency in SGR before the start of the constant light period. That is, after being exposed to the stress regime, UCS groups had very low values (0.47) compared with control groups (1.05). UCS fish had overall higher SGR values during smoltification (sampling 2) and one month after seawater transfer (sampling 3), compared with control fish (figure 2*c*).

### 3.3. Monoamine neurochemistry

**Figure 3.**
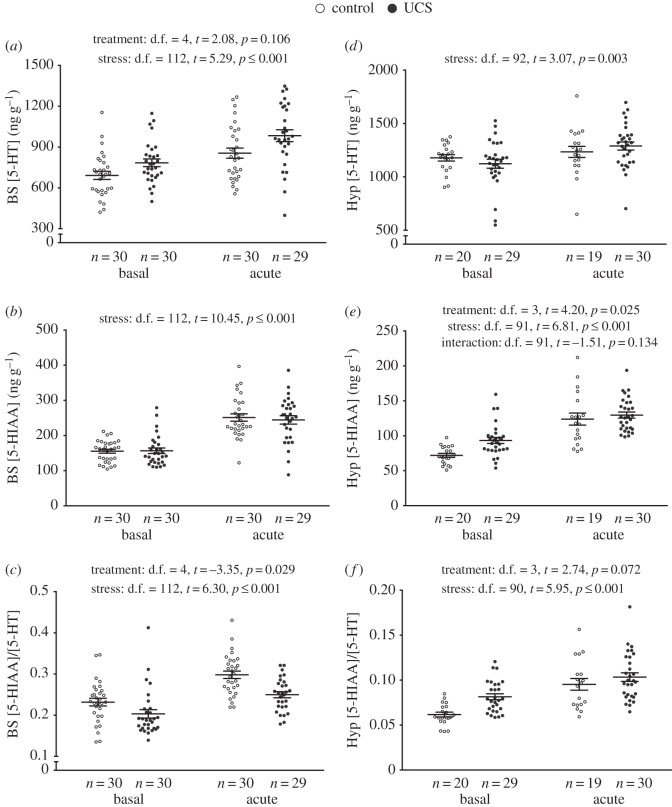
Effect of stress treatment (i.e. chronic unpredictable stress (UCS) or control) at basal and acute-stress conditions on serotonin (5-HT) neurochemistry in the brain stem (BS) (*a*–*c*) and hypothalamus (Hyp) (*d*–*f*) of Atlantic salmon. Linear model effect statistics are given in figure for each panel.

*Serotonergic neurochemistry*. Irrespective of treatment, 5-HT, its main catabolite 5-HIAA levels, and the 5-HIAA/5-HT ratio increased after acute stress in both brain areas (figure 3). A general treatment effect in the brain stem was found for the 5-HIAA/5-HT ratio, whereby control groups had significantly higher 5-HIAA/5-HT values compared with the UCS fish (figure 3*c*). In addition, the UCS fish had significantly higher 5-HIAA levels in the hypothalamus (figure 3*e*), with a tendency for higher 5-HIAA/5-HT ratios (figure 3*f*).

**Figure 4.**
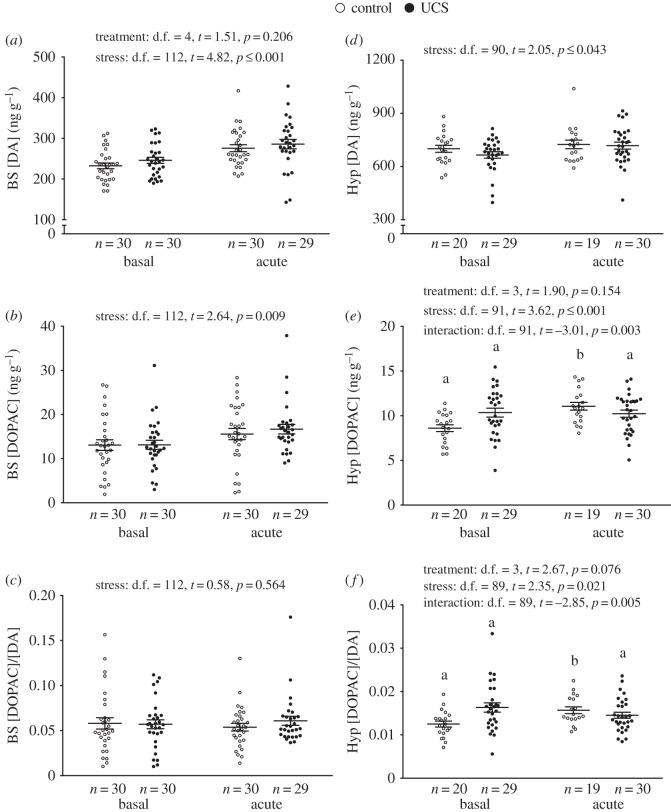
Effect of stress treatment (i.e. unpredictable chronic stress (UCS) or control) at basal and acute-stress conditions on dopamine (DA) neurochemistry in the brain stem (BS) (*a*–*c*) and hypothalamus (Hyp) (*d*–*f*) of Atlantic salmon. Linear model effect statistics are given in figure for each panel. Lowercase letters indicate a significant stress effect within treatment.

*Dopaminergic neurochemistry*. Irrespective of treatment, brain stem DA and its main catabolite DOPAC both significantly increased in response to stress (figure 4*a*,*b*), but there was no effect on the DOPAC/DA ratio (figure 4*c*). In the hypothalamus, there was a significant increase in both DOPAC levels and the DOPAC/DA ratio in response to stress in controls, but not in UCS fish (figure 4*e*,*f*).

**Figure 5.**
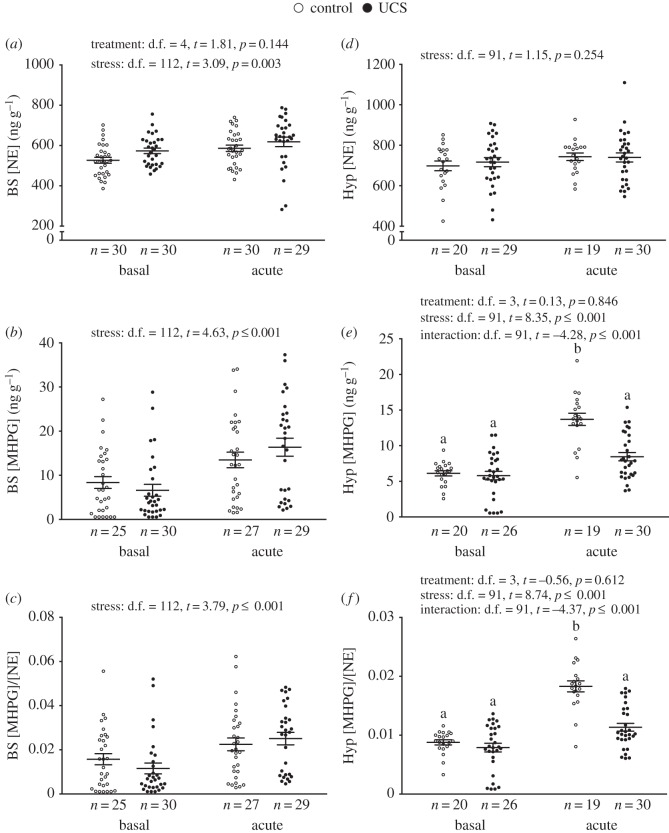
Effect of stress treatment (i.e. unpredictable chronic stress (UCS) or control) at basal and acute-stress conditions on noradrenaline (NE) neurochemistry in the brain stem (BS) (*a*–*c*) and hypothalamus (Hyp) (*d*–*f*) of Atlantic salmon. Linear model effect statistics are given in figure for each panel. Lowercase letters indicate a significant treatment effect within stress groups.

*Noradrenaline neurochemistry*. Irrespective of treatment, NE, its main catabolite MHPG and the MHPG/NE ratio in the brain stem significantly increased after acute stress (figure 5*a*–*c*). There was a significant interaction effect in both hypothalamic MHPG levels and the MHPG/NE ratio, where control fish only had a significantly higher response post-stress, but not UCS groups (figure 5*e*,*f*).

### 3.4. Correlation analysis

**Figure 6.**
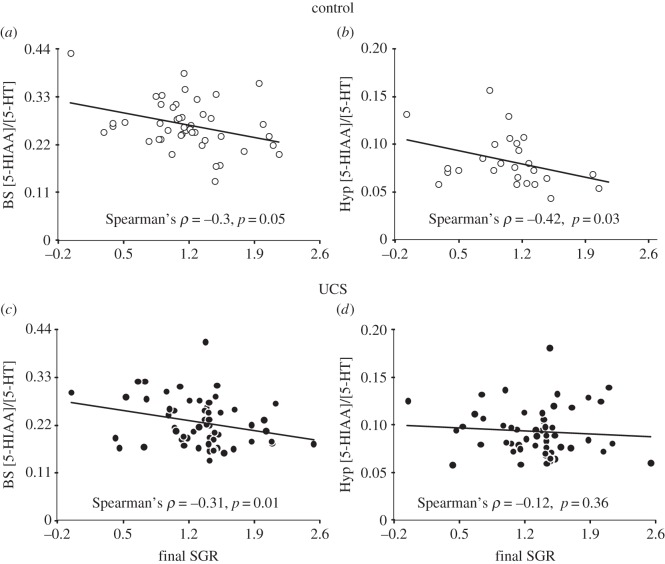
Correlation between mean (±s.e.m.) specific growth rate (SGR) and 5-HIAA/5-HT ratios for pooled basal and acute-stress conditions in the brain stem (left) and hypothalamus (right) in control (*a*,*b*) and unpredictable chronic stress (UCS) (*c*,*d*) groups. Spearman's *c*orrelation analysis values are presented in figure for each panel.

We analysed pooled data for acute and basal conditions for all groups using non-parametric Spearman rank correlation analysis to investigate possible relationships between growth rate at the end of the experiment and the 5-HIAA/5-HT ratios in both studied brain areas. Control fish displayed a significant negative correlation between these variables in both brain areas (*Spearman's ρ* = −0.3, *p* = 0.05 and −0.42, *p* = 0.03, figure 6*a,b* for brain stem and hypothalamus, respectively). UCS fish also showed a significant negative correlation in the brain stem (*Spearman's ρ* = −0.31, *p* = 0.01; figure 6*c*), but this was not the case in the hypothalamus (*Spearman's ρ* = −0.12, *p* = 0.36, figure 6*d*).

## 4. Discussion

Here, we show evidence that Atlantic salmon subjected to a stressful regime during early life display a mitigated hypothalamic catecholaminergic (CA) and brain stem serotonergic response to acute stress later in life, compared to non-treated fish. Furthermore, the growth rate of the early stress-treated salmon was higher than those of control fish after seawater adaption and transfer, life stages that are particularly stressful for salmonids [16,17]. Taken together these results show how early life stress treatment has long-term consequences in the way individuals respond to their environment later in life (i.e. phenotypic plasticity). Interestingly, it has been proposed that phenotypic plasticity in response to stress in a new environment may ultimately reflect a fundamental breakdown of physiological function. That is, responses to stress may become non-adaptive in environments that differ from those encountered by the ancestral phenotype [18], such as salmon in aquaculture. However, plasticity may become adaptive if the phenotype produced is the one favoured by selection in the new environment [18]. In the case of salmonid aquaculture, artificial selection has favoured stress-resilient phenotypes [19,20] and therefore, it could be tempting to speculate that experiencing stress (at key life stages) could induce a certain level of habituation (e.g. a mitigated monoamine response) and therefore increase the overall fitness within the population. Alternatively, this mitigated response may be a consequence of allostatic overload, the inability of regulatory mechanisms to react to further challenges [21], and represent a non-adaptive response. We discuss our data in this context.

In the UCS groups, we used stressors unpredictable in nature, representing a higher aversive challenge than a single predictable stressor [12]. This resulted in poorer body condition at the end of the stress regime, and a general tendency for lower growth rates in UCS groups (approx. 0.47) compared with control (approx. 1.05). This is most probably explained by stress, which reduces appetite, but increases metabolism in fishes [22,23]. This is important because unpredictable (in time and/or space) stressful stimuli are considered more severe than predictable stressors [1,2]. Notably, it has been proposed that exposure to unpredictable stress during early life stages may help individuals cope better later in life to harsh/stressful environments [2]. This might be particularly important in the aquaculture environment, as normal husbandry practices may represent both predictable and unpredictable challenges for fish in time and form. That is, fish may be handled at working hours for several different practices, such as vaccination, grading, transport, etc. and this may happen at different times throughout the day/season [24]. Therefore, it is necessary for fish to adapt to unpredictability in order to thrive in this environment. Notably, during seawater adaption, a process known as smoltification, salmonids deplete their fat reserves [16,17], which is illustrated by lower body condition [14], as was the case in our experiment for both groups. However, the UCS fish had a significantly higher growth rate during smoltification and after seawater transfer compared with control fish, which indicates a better use of resources during these time-periods and a compensatory growth rate in UCS groups, as has been reported before in fish, which have experienced harsh conditions [25–27]. Notably, the seawater stage in the present experiment did not replicate the full magnitude of change commonly encountered by salmonids in aquaculture. That is, we exposed our fish to seawater within their housing tanks, whereas commercially produced salmonids are typically transported from land based rearing facilities into floating sea cages. This transport procedure is known to be particularly stressful [28]. In addition, the commercial situation also results in environmental changes, with larger volume and depth, different light conditions, and altered feeding procedures [24]. Therefore, it would be interesting to test UCS and control groups using typical aquaculture practices in order to evaluate further the growth performance by UCS fish after seawater transfer.

When assessing brain monoaminergic activity, it is common to use either the catabolite concentration itself, or the ratio of the catabolite to the neurotransmitter, as a biochemical proxy of neuronal activity (catabolites being formed chiefly after release and re-uptake of the parent monoamine, e.g. for serotonin: [5-HIAA]/[5-HT] or [5-HIAA] only) [29]. In this experiment, we found that while all groups responded with increased serotonergic activity to acute stress, this response was significantly higher in the brain stem of control fish. In addition, we found an overall tendency for CA, i.e. DA and NE, systems to increase their activity after acute stress in the brain stem of all groups, while only control individuals showed a significant post-stress increase in hypothalamic dopaminergic and noradrenergic activity. Taking all results together, we find that hypothalamic CA and brain stem serotonergic activity in UCS groups post-stress appears to be mitigated, compared to control groups.

In the vertebrate brain, 5-HT has a crucial role in energy regulation, neural plasticity, behavioural and emotional control, as well as neuroendocrine responses to stress [30,31]. In the fish brain, serotonergic activity has been found to consistently increase in response to stress [32–35]. In terms of energy regulation, it has been proposed that 5-HT signalling increases in conditions that require the reallocation of energy resources. Therefore, stressful events would increase serotonergic activity and reallocate energy from processes such as growth and reproduction towards cognition and coping behaviours [31]. Notably, as mentioned above, smoltification is a very energy demanding process and it is associated with an overall 50% increase in brain 5-HT [36] as well as a 100% increase in cortisol levels [37], which are both associated with increased catabolism of energy reserves. Furthermore, chronically stressed salmonids (e.g. subordinate fish) show lower growth rates than non-stressed ones and are also characterized by increased brain 5-HT activity [38]. Furthermore, 5-HT has been shown to have an inhibitory effect on the release of growth hormone [39]. In other words, there is a general tendency for reduced growth in individuals displaying high 5-HT activity. Presently, we have found a negative correlation between the final SGR and serotonergic activity in the brain stem for all individuals, and for control fish only, in the hypothalamus. That is, individuals that displayed higher serotonergic activity in response to stress had overall lower growth than less reactive individuals. This has been previously reported to be indicative of rank within a social hierarchy, with small individuals having a lower rank [40]. Interestingly, it has been proposed that the serotonergic system regulates energy metabolism through several pathways including the regulation in the production of ATP from glucose by stimulating the breakdown of glycogen from astrocytes via 5-HT_1A_ heteroreceptors, the regulation of glucagon and insulin secretion from pancreatic cells, the regulation of stored body fat through leptin signalling pathways, the control of the energetic resources through vasodilation and a bidirectional control of neuronal activity (neurons are major consumers of energy in the brain, for a review see [31]). Therefore, further experiments are needed to clarify the potential involvement of 5-HT in the neuroendocrine mechanisms underlying the growth differences between early life stress and non-stressed controls. In this context, smoltification appears to be especially interesting, because this is associated with changes in monoaminergic signalling and a mitigated monoaminergic response during this oncogenic shift may be part of the mechanism behind the increased growth displayed by UCS fish in this study.

CA systems are believed to be fundamental in the variation of behavioural flexibility through stimuli salience regulation, and their role in attention, perception and impulse control [41–44]. Notably, high levels of DA and NE have been associated with increased arousal during novel stressful situations [45,46]. The organization of monoamine systems is intricate and includes complex interactions in the regulation of key-brain functions, such as cognition, motor-function and emotions [47]. Together, the activity of monoaminergic systems helps integrate internal physiological demands dependent upon how environmental input is processed (i.e. environmental and physiological inputs are interpreted as a function of context and not in a generalized manner). This shapes how animals behave and regulate their physiological processes [43,44,47,48]. Our results show that UCS fish either recover faster (because measurements of monoamine activity were taken 1 h after acute stress) or have a mitigated response to stress. That is, as we only measured onetime point, it is not possible to determine if control individuals reacted with higher monoaminergic levels to stress or if UCS groups had already recovered from the stress response and have therefore lower levels at this time point. Nevertheless, UCS groups showed overall lower hypothalamic CA and brain stem serotonergic levels post-stress compared with controls. This may imply that UCS groups may be partially habituated to stress and are therefore more capable to reallocate resources from stress coping into other life processes, compared to more stress-naive individuals. Alternatively, cumulative stress may overload physiological systems and compromise their ability to react further to stressors (i.e. allostatic overload [21]). It would therefore be particularly interesting to study these groups over longer periods, including several months after transfer to sea cages (with all the stressors this implies, as explained above). Notably, it has been reported that up to 25% of fish in aquaculture farms display a depression-like state (DLS) [49] and most of them are later lost, owing to their inability to cope after seawater transfer [15,50]. As an overload of cumulative stress has been associated with depressive states [2], it would be of particular interest to study how a stress regime during early life could reduce or promote the occurrence of DLS phenotypes in aquaculture.

In conclusion, we found that Atlantic salmon which experienced early life stress (UCS), display a higher growth rate during two challenging developmental periods: during smoltification and after seawater transfer. Furthermore, one month after seawater transfer UCS groups had a mitigated hypothalamic CA and brain stem serotonergic response to stress. Our results indicate that individuals who experience early life stress respond differently to environmental stimuli later in life (up to 10 weeks after the stress regime) compared with non-stress-treated fish. This is in agreement with the belief that physiological and behavioural responses represent trade-offs from life-history strategies and should be viewed/interpreted in a context-dependent manner [2]. That is, in similar studies [12,51,52], it has been concluded that an ablated/mitigated response to stress is a negative consequence of cumulative stress (i.e. allostatic overload), which might be true in some situations. Alternatively, we propose that in an aquaculture-environmental context, where stressful situations are common, experiencing stress from an early age may help individuals cope better with their environment later in life. This is in line with the allostasis theory [21], which proposes that individuals which repeatedly experienced challenges are better equipped to cope with future similar stressors. For example, by mounting a lesser monoaminergic stress response, individuals may be able to invest more energy into other life processes, such as growth. We hope that future studies will be focused towards better understanding of allostatic processes and both the possible negative and positive consequences of early life stress in a context-dependent manner.

## Supplementary Material

Data file

## Data accessibility

All relevant data are within the paper and its electronic supplementary material.
